# Supplementary material for: Conformational and dynamic plasticity in substrate-binding proteins underlies selective transport in ABC importers
Source: eLife. 2019 Mar 22;8:e44652. doi: 10.7554/eLife.44652 (PMC6450668; doi:10.7554/eLife.44652)
Supplement: Supplementary file 1. [file elife-44652-supp1.docx]

* *P* < 10^-10^

| **MalE(T36C/S352C)** | apo | Β-cyclo-dextrin | Malto-triitol | Malto-tetraitol | Malto-  heptaose | Malto-octaose | Malto-decaose | Malto-hexaose | Malto-pentaose | Maltose | Malto-  triose | Malto-  tetraose |
| --- | --- | --- | --- | --- | --- | --- | --- | --- | --- | --- | --- | --- |
| apo |  | * |  |  |  |  |  |  |  |  |  |  |
| Β-cyclodextrin |  |  | * | * |  |  |  |  |  |  |  |  |
| maltotriitol |  |  |  | 0.68 |  |  |  |  |  |  |  |  |
| maltotetraitol |  |  |  |  | * | * |  |  |  |  |  |  |
| maltoheptaose |  |  |  |  |  | 0.93 | 0.60 | * |  |  |  |  |
| maltooctaose |  |  |  |  |  |  | 0.47 | * |  |  |  |  |
| maltodecaose |  |  |  |  |  |  |  | * | * | * |  |  |
| maltohexaose |  |  |  |  |  |  |  |  | 0.94 | * | * | * |
| maltopentaose |  |  |  |  |  |  |  |  |  | * | * | * |
| maltose |  |  |  |  |  |  |  |  |  |  | 0.68 | 0.57 |
| maltotriose |  |  |  |  |  |  |  |  |  |  |  | 0.43 |
| maltotetraose |  |  |  |  |  |  |  |  |  |  |  |  |

| **MalE(T36C/N205C)** | apo | maltoheptaose | maltooctaose | maltodecaose |
| --- | --- | --- | --- | --- |
| apo |  | * | * | * |
| maltoheptaose |  |  | 0.29 | 0.89 |
| maltooctaose |  |  |  | 0.13 |
| maltodecaose |  |  |  |  |

| **MalE(K34C/R354C)** | apo | maltoheptaose | maltooctaose | maltodecaose |
| --- | --- | --- | --- | --- |
| apo |  | * | * | * |
| maltoheptaose |  |  | 0.95 | 0.90 |
| maltooctaose |  |  |  | 0.93 |
| maltodecaose |  |  |  |  |

| **OpuAC(A209C/S441C**) | Carnitine | apo | proline | Glycine betaine |
| --- | --- | --- | --- | --- |
| Carnitine |  | * |  |  |
| Apo |  |  | * | * |
| proline |  |  |  | * |
| Glycine betaine |  |  |  |  |

| **PsaA(V76C/K237C)** | Apo | Mn^2+^ | Zn^2+^ |
| --- | --- | --- | --- |
| Apo |  | * | * |
| Mn^2+^ |  |  | 0.21 |
| Zn^2+^ |  |  |  |

| **PsaA(E74C/K237C)** | Apo | Mn^2+^ | Zn^2+^ |
| --- | --- | --- | --- |
| Apo |  | * | * |
| Mn^2+^ |  |  | 0.16 |
| Zn^2+^ |  |  |  |

| **SBD2(T369C/S451)** | Apo | Arginine | Lysine | Glutamine | Asparagine |
| --- | --- | --- | --- | --- | --- |
| Apo |  | 0.99 | 0.89 | * | * |
| Arginine |  |  | 0.45 | * |  |
| Lysine |  |  |  | * |  |
| Glutamine |  |  |  |  | * |
| Asparagine |  |  |  |  |  |

| **SBD1(G87C/T159C)** | Apo | Arginine | Glutamate | Glutamine | Histidine |
| --- | --- | --- | --- | --- | --- |
| Apo |  | 0.75 | * | * | * |
| Arginine |  |  |  |  |  |
| Glutamate |  |  |  | * | * |
| Glutamine |  |  |  |  | * |
| Histidine |  |  |  |  |  |

| **OppA(A209C/S441C)** | apo | RPPGFSPFR | RDMPIQAF | SLSQSKVLPVPQ | SLSQSKVLP |
| --- | --- | --- | --- | --- | --- |
| Apo |  | * | * | * | * |
| RPPGFSPFR |  |  | 0.64 | 0.35 | 0.49 |
| RDMPIQAF |  |  |  | 0.14 | 0.89 |
| SLSQSKVLPVPQ |  |  |  |  | 0.06 |
| SLSQSKVLP |  |  |  |  |  |
