# Supplementary material for: Conformational and dynamic plasticity in substrate-binding proteins underlies selective transport in ABC importers
Source: eLife. 2019 Mar 22;8:e44652. doi: 10.7554/eLife.44652 (PMC6450668; doi:10.7554/eLife.44652)
Supplement: Supplementary file 2. [file elife-44652-supp2.docx]

**Primers used in this study**

| Protein | Primer (5’ to 3’) |
| --- | --- |
| Forward PsaA(V76C) | CAAATCAGCCTCAGAAGTTTTCTTGCAGTCTTCAGGAAGTGGTTCGTATTC |
| Reverse PsaA(V76C) | GAATACGAACCACTTCCTGAAGACTGCAAGAAAACTTCTGAGGCTGATTTG |
| Forward PsaA(K237C) | GGCGAAGTTTTTCAACCAAGGTGCAGATTTGTTCAGGAGTTCCTTCT |
| Reverse PsaA(K237C) | AGAAGGAACTCCTGAACAAATCTGCACCTTGGTTGAAAAACTTCGCC |
| Forward OpuAC(V360C) | ACAGCTTTAGATAATGCGTGTGCTTGGCAAACAGTAGCC |
| Reverse OpuAC(V360C) | GGCTACTGTTTGCCAAGCACACGCATTATCTAAAGCTGT |
| Forward OpuAC(N423C) | TCAATTGAAGATTTAACATGTCAAGCGAATAAAACAATC |
| Reverse OpuAC(N423C) | GATTGTTTTATTCGCTTGACATGTTAAATCTTCAATTGA |
| Reverse OppA(A209C) | TGTCGTTTTTGGACTAGAACACAAATCTTTAGGAGCGAC |
| Forward OppA(S441C) | AAAATTGGGGTAAAAGTGTGTCTTTATAACGGTAAATTG |
| Forward MalE(T36C) | GATACCGGAATTAAAGTCTGCGTTGAGCATCCGGATAAA |
| Reverse MalE(T36C) | TTTATCCGGATGCTCAACGCAGACTTTAATTCCGGTATC |
| Forward MalE(S352C) | GTGATCAACGCCGCCTGCGGTCGTCAGACTGTC |
| Reverse MalE(S352C) | GACAGTCTGACGACCGCAGGCGGCGTTGATCAC |
| Forward MalE(N205C) | ATTAAAAACAAACACATGTGCGCAGACACCGATTACTCC |
| Reverse MalE(N205C) | GGAGTAATCGGTGTCTGCGCACATGTGTTTGTTTTTAAT |
| Forward MalE(K34C) | GAGAAAGATACCGGAATTTGCGTCACCGTTGAGCATCCG |
| Reverse MalE(K34C) | CGGATGCTCAACGGTGACGCAAATTCCGGTATCTTTCTC |
| Forward MalE(R354C) | AACGCCGCCAGCGGTTGCCAGACTGTCGATGAA |
| Reverse MalE(R354C) | TTCATCGACAGTCTGGCAACCGCTGGCGGCGTT |
| Forward MalE(A96W) | TATCCGTTTACCTGGGATTGGGTACGTTACAACGGCAAG |
| Reverse MalE(A96W) | CTTGCCGTTGTAACGTACCCAATCCCAGGTAAACGGATA |
| Forward MalE(I329W) | AACGCCCAGAAAGGTGAATGGATGCCGAACATCCCGCAG |
| Reverse MalE(I329W) | CTGCGGGATGTTCGGCATCCATTCACCTTTCTGGGCGTT |
| Forward MalE isolation | GGGAATTCCATATGAAA ATCGAAGAAGGTAAACTGGTAATCTGG |
| Forward MalE isolation | GACCCGAAGCTTCTTGGTGATACGAGTCTGCGCGTCTTTCAGGGCTTC |
| psaA_F | GGAAAAAAAGATACAACTTCTGGTC |
| psaA_R | TTATTTTGCCAATCCTTCAG |
| psaA_X | GACCAGAAGTTGTATCTTTTTTTCC |
| psaA_Y | CTGAAGGATTGGCAAAATAA |
| psaA_Flank_F | CTGGTCTAAATCAACAAAACCTC |
| psaA_Flank_R | GACCTATAGCTTACTAGCTCTTGTCTT |
| czcD_Flank_F | GAGCCCAATTTCGTCTGGG |
| czcD_Flank_R | TAGCTATCGGTGCCCTCCG |
| czcD_F | ATGAAGGCAAAATATGCTGTTTG |
| czcD_R | CTAATGTTGATGCTCATAACTCCG |
| czcD_X | CAAACAGCATATTTTGCCTTCAT |
| czcD_Y | CGGAGTTATGAGCATCAACATTAG |
| czcD_Janus_X | CATTATCCATTAAAAATCAAACGGTATTCAGTTCTGAACAATTTGCC |
| czcD_Janus_Y | GGAAAGGGGCCCAGGTCTCTGTGAAAAATACTTGGGTACTATCTT |
| psaAD280N_F | cccaatctacgcacaaatctttactaactctatcgcaga |
| psaAD280N_R | tctgcgatagagttagtaaagatttgtgcgtagattggg |
