# Supplementary material for: Conformational and dynamic plasticity in substrate-binding proteins underlies selective transport in ABC importers
Source: eLife. 2019 Mar 22;8:e44652. doi: 10.7554/eLife.44652 (PMC6450668; doi:10.7554/eLife.44652)
Supplement: Supplementary file 10 [file elife-44652-supp3.odt]

Apparent FRET efficiency values of solution-based measurements

| #### Proteina | Condition | Apparent FRET | Inter-dye  distance change (Å)b | Cα-Cα distance in crystal structure (Å)b,c |
| --- | --- | --- | --- | --- |
| OpuAC(V360C/N423C) | no ligand | 0.501 ± 0.004 |  |  |
|  | carnitine | 0.479 ± 0.003 | -1.2 ± 0.3 |  |
|  | proline | 0.543 ± 0.004 | 3.4 ± 0.3 |  |
|  | glycine betaine | 0.646 ± 0.003 | 9.0 ± 0.3 | 9.4 |
| #### SBD2(T369C/S451) | no ligand | 0.492 ± 0.003 |  |  |
|  | #### glutamate | 0.633 ± 0.004 | 7.2 ± 0.4 |  |
|  | #### glutamine | 0.677 ± 0.002 | 9.4 ± 0.4 | 9.3 |
|  | #### arginine | 0.496 ± 0.002 | 0.0 ± 0.3 |  |
| #### SBD1(G87C/T159C) | #### no ligand | 0.612 ± 0.003 |  |  |
|  | #### glutamine | 0.710 ± 0.003 | 4.6 ± 0.6 |  |
|  | asparagine | 0.805 ± 0.002 | 9.4 ± 1.2 |  |
|  | #### histidine | 0.761 ± 0.003 | 7.2 ± 1.0 |  |
|  | arginine | 0.610 ± 0.002 | 0.0 ± 0.4 |  |
|  | #### lysine | 0.613 ± 0.002 | 0.0 ± 0.4 |  |
| #### MalE(T36C/S352C) | no ligand | 0.646 ± 0.003 |  |  |
|  | #### Β-cyclodextrin | 0.696 ± 0.003 | 2.7 ± 0.2 |  |
|  | #### maltotriitol | 0.725 ± 0.003 | 3.7 ± 0.2 |  |
|  | #### maltotetraitol | 0.725 ± 0.003 | 3.7 ± 0.2 |  |
|  | #### maltose | 0.824 ± 0.002 | 9.8 ± 0.3 | 10.2 |
|  | #### maltotriose | 0.826 ± 0.003 | 9.7 ± 0.3 |  |
|  | #### maltotetraose | 0.824 ± 0.002 | 9.6 ± 0.3 |  |
|  | #### maltopentaose | 0.777 ± 0.004 | 6.8 ± 0.3 |  |
|  | #### maltohexaose | 0.779 ± 0.003 | 6.8 ± 0.2 |  |
|  | #### maltoheptaose | 0.746 ± 0.002 | 5.2 ± 0.2 |  |
|  | maltooctaose | 0.745 ± 0.003 | 5.1 ± 0.2 |  |
|  | maltodecaose | 0.742 ± 0.003 | 5.2 ± 0.2 |  |
| #### MalE(T36C/N205C) | #### no ligand | 0.538 ± 0.007 |  |  |
|  | #### maltoheptaose | 0.755 ± 0.005 | 6.4 ± 0.5 |  |
|  | #### maltooctaose | 0.758 ± 0.005 | 6.5 ± 0.5 |  |
|  | #### maltodecaose | 0.757 ± 0.006 | 6.4 ± 0.5 |  |
| #### MalE(K34C/R354C) | #### no ligand | 0.524 ± 0.010 |  |  |
|  | #### maltoheptaose | 0.669 ± 0.008 | 8.7 ± 0.6 |  |
|  | #### maltooctaose | 0.662 ± 0.007 | 8.8 ± 0.6 |  |
|  | #### maltodecaose | 0.666 ± 0.008 | 8.8 ± 0.6 |  |
| #### OppA(A209C/S441C) | #### no ligand | 0.621 ± 0.004 |  |  |
|  | #### RPPGFSPFR | 0.803 ± 0.003 | 9.3 ± 0.4 | 12.7 |
|  | #### RDMPIQAF | 0.803 ± 0.003 | 9.2 ± 0.4 |  |
|  | #### SLSQSKVLPVPQ | 0.807 ± 0.004 | 9.3 ± 0.4 |  |
|  | #### SLSQSKVLP | 0.801 ± 0.003 | 9.3 ± 0.4 |  |
| PsaA(V76C/K237C) | no ligand | 0.615 ± 0.003 |  |  |
|  | Mn2+ | 0.681 ± 0.004 | 4.0 ± 0.4 | 3.8 |
|  | Zn2+ | 0.688 ± 0.004 | 4.1 ± 0.4 |  |
| PsaA(E74C/K237C) | no ligand | 0.518 ± 0.003 |  |  |
|  | Mn2+ | 0.567 ± 0.003 | 3.2 ± 0.3 | 3.5 |
|  | Zn2+ | 0.570 ± 0.003 | 3.2 ± 0.3 |  |
| PsaA(D280N/V76C/K237C) | no ligand | 0.617 ± 0.003 |  |  |
|  | Zn2+ | 0.689 ± 0.005 | 4.1 ± 0.4 |  |

- Only data of the same protein construct can be compared (all recorded on one day), due to unavoidable differences in microscope settings when measurements are done on different days. Qualitatively consistent results were obtained upon repetition with an independent labeled protein sample and measured on a different day.

- Error denotes 95% confidence interval for the mean.

- Distance calculated from crystal structures with PDB codes: OpuAC (3L6G, 3L6H), SBD2 (4KR5, 4KQP), PsaA (3ZK7, 1PSZ), OppA (3FTO, 3DRG) and MalE (1OMP, 1ANF).
