# Supplementary material for: Conformational and dynamic plasticity in substrate-binding proteins underlies selective transport in ABC importers
Source: eLife. 2019 Mar 22;8:e44652. doi: 10.7554/eLife.44652 (PMC6450668; doi:10.7554/eLife.44652)
Supplement: Supplementary file 11 [file elife-44652-supp4.odt]

Statistics of confocal scanning experiments of immobilized molecules.

| Proteina | Conditionb | Molecules analysed | Total observation time (min) | Transitions observed |
| --- | --- | --- | --- | --- |
| OpuAC(V360C/N423C) | no ligand + 20 µM protein | 702 | 8.2 | 0 |
|  | 3.9 µM glycine betaine | 102 | 1.2 | 2692 |
|  | 1 mM glycine betaine | 94 | 2.3 | 0 |
| OppA(A209C/S441C) | no ligand + 2 µM protein | 202 | 6.4 | 21 |
|  | no ligand + 10 µM protein | 311 | 6.6 | 26 |
|  | 2.5 µM RPPGFSPFR | 43 | 1.4 | 267 |
|  | 5 µM RPPGFSPFR | 168 | 4.6 | 1080 |
|  | 10 µM RPPGFSPFR | 143 | 4.5 | 1800 |
|  | 20 µM RPPGFSPFR | 76 | 2.2 | 1683 |
|  | 200 µM RPPGFSPFR | 31 | 0.5 | 0 |
| SBD1(G87C/T159C) | no ligand + 1 µM protein | 133 | 6.2 | 18 |
|  | no ligand + 20 µM protein | 292 | 20.6 | 50 |
|  | 200 nM asparagine | 22 | 1.5 | 207 |
|  | 50 µM asparagine | 72 | 8.0 | 0 |
| SBD2(T369C/S451) | no ligand + 4 µM protein | 148 | 6.1 | 51 |
|  | no ligand + 16 µM protein | 166 | 5.1 | 38 |
|  | 200 nM glutamine | 23 | 1.8 | 498 |
|  | 50 µM glutamine | 82 | 1.0 | 0 |
| MalE(T36C/S352C) | no ligand + 20 µM protein | 503 | 10.9 | 0 |
|  | 1 mM maltotetraose | 32 | 1.2 | 0 |
|  | 1.2 µM maltose | 37 | 1.6 | 378 |
|  | 400 nM maltotriose | 144 | 7.2 | 968 |
|  | 450 nM maltotetraose | 70 | 1.8 | 345 |
|  | 2 µM maltoheptaose | 61 | 4.9 | 591 |
|  | 2 µM maltooctaose | 50 | 2.2 | 491 |
|  | 4 µM maltodecaose | 75 | 4.9 | 257 |
| MalE(T36C/S352C/A96W/I329W) | 10 nM maltose | 20 | 20.4 | 30 |
| PsaA(76C/K237C) | no ligand + 1 mM EDTA | 254 | 26.3 | 0 |
|  | 200 µM Mn2+ | 35 | 5.8 | 0 |

- Fluorescence trajectories of 2 independent labeled proteins samples where pooled and analyzed.

- To scavenge potential ligand contaminations µM concentrations of unlabeled protein was added to study apo protein (details in text).
